# Locoregional therapy combined with systemic therapy (LRT + ST) for unresectable and metastatic intrahepatic cholangiocarcinoma: a systematic review and meta-analysis

**DOI:** 10.2478/raon-2023-0059

**Published:** 2023-11-30

**Authors:** Mengqi Zhang, Weiwei Qi, Xiaofei Qiu, Chunpeng Yu, Wensheng Qiu, Song Wang, Zhenkang Qiu

**Affiliations:** Department of Oncology, Key Laboratory of Cancer Molecular and Translational Research, The Affiliated Hospital of Qingdao University, Qingdao, Shandong, China; Interventional Medical Center, The Affiliated Hospital of Qingdao University, Qingdao, Shandong, China; Qingdao Municipal Center for Disease Control & Prevention, Qingdao Institute of Preventive Medicine, Qingdao, Shandong, China

**Keywords:** unresectable iCCA, locoregional therapy, systemic therapy, meta-analysis

## Abstract

**Background:**

The outcome of systemic therapy (ST) for unresectable and metastatic intrahepatic cholangiocarcinoma (iCCA) is poor. This study aims to further evaluate the efficacy and safety of locoregional therapy combined with systemic therapy (LRT + ST) compared with only ST in unresectable and metastatic iCCA by performing a systematic literature review and meta-analysis.

**Methods:**

A comprehensive search was performed in PubMed, Web of Science, EMBASE, and the Cochrane Library up to November 3, 2022. The primary outcome was overall survival (OS), and the secondary outcomes were progression-free survival (PFS), objective response rate (ORR), and adverse events (AEs).

**Results:**

Ten retrospective cohort studies with 3,791 unresectable or metastatic iCCA patients were enrolled in this study, including 1,120 who received ablation, arterially directed therapy (ADT), or external beam radiation therapy (EBRT) combined with ST. The meta-analysis showed that the LRT + ST group had a better OS (*HR* = 0.51; *95% CI* =0.41–0.64; *p value* < 0.001), PFS (*HR* = 0.40, *95% CI* = 0.22–0.71, *p value* = 0.002) and ORR (*RR* = 1.68; *95% CI* = 1.17–2.42; *p value* = 0.005). Subgroup analysis showed that both ST combined with ADT (*HR* = 0.42, *95% CI* = 0.31–0.56, *p value* < 0.001) and EBRT (*HR* = 0.67, *95% CI* = 0.63–0.72, *p value* < 0.001) could improve OS. Neutropenia, thrombocytopenia, anemia, anorexia, and vomiting did not show significant differences between the groups (p value > 0.05).

**Conclusions:**

Compared with only ST, LRT + ST improved survival outcomes for unresectable and metastatic iCCA patients without increasing severe AEs, which can further provide a basis for guidelines.

## Introduction

Intrahepatic cholangiocarcinoma (iCCA), which develops from the bile duct within the hepatic parenchyma, has been increasing in incidence and mortality in recent years.^1,2,3^ Due to the absence of symptoms at the initial stage, only approximately 22% of iCCA patients are resectable at the primary diagnosis.^4^ The ABC-02 trial established gemcitabine plus cisplatin (GemCis) as the first-line treatment for locally advanced or metastatic biliary tract cancer, with a median overall survival (OS) was 11.7 months.^5^ The recent TOPAZ-1 trial demonstrated that using durvalumab plus GemCis improved patient survival compared with GemCis alone, bringing a median survival benefit of 1.3 months compared to GemCis.^6^

With the rapid development of equipment and technology, locoregional therapies, such as ablation^7,8^, arterially directed therapies (ADTs)^9,10,11,12,13^, and external beam radiation therapy (EBRT)^14,15,16,17^, have shown excellent effects on iCCA. However, there have been conflicting opinions about the effectiveness and safety of locoregional therapy combined with systemic therapy (LRT + ST) in unresectable or metastatic iCCA because of the absence of randomized controlled trials or meta-analyses. Evaluating this combination therapy method's effectiveness, long-term survival, and safety is difficult because most evidence is derived from retrospective cohort studies, case reports, or series. Thus, this study was performed to explore the feasibility and survival benefits of LRT + ST for unresectable and metastatic iCCA via a systematic review and meta-analysis.

## Material and methods

This systematic review and meta-analysis were performed according to the Preferred Reporting Items for Systematic Reviews and Meta-Analyses (PRISMA) guidelines^18^ and registered in PROSPERO (registration: CRD42020162902).

### Literature search

A literature search was conducted in PubMed, Web of Science, EMBASE, and the Cochrane Library on November 3, 2022, without language or geographic restrictions. The search terms included “unresectable intrahepatic cholangiocarcinoma”, “locoregional therapy”, “systemic therapy”, and their synonyms. The detailed search strategies are provided in Supplementary Table 1. The reference lists of the final included studies were also checked for possible additional records.

### Study selection and eligibility criteria

**FIGURE 1.**
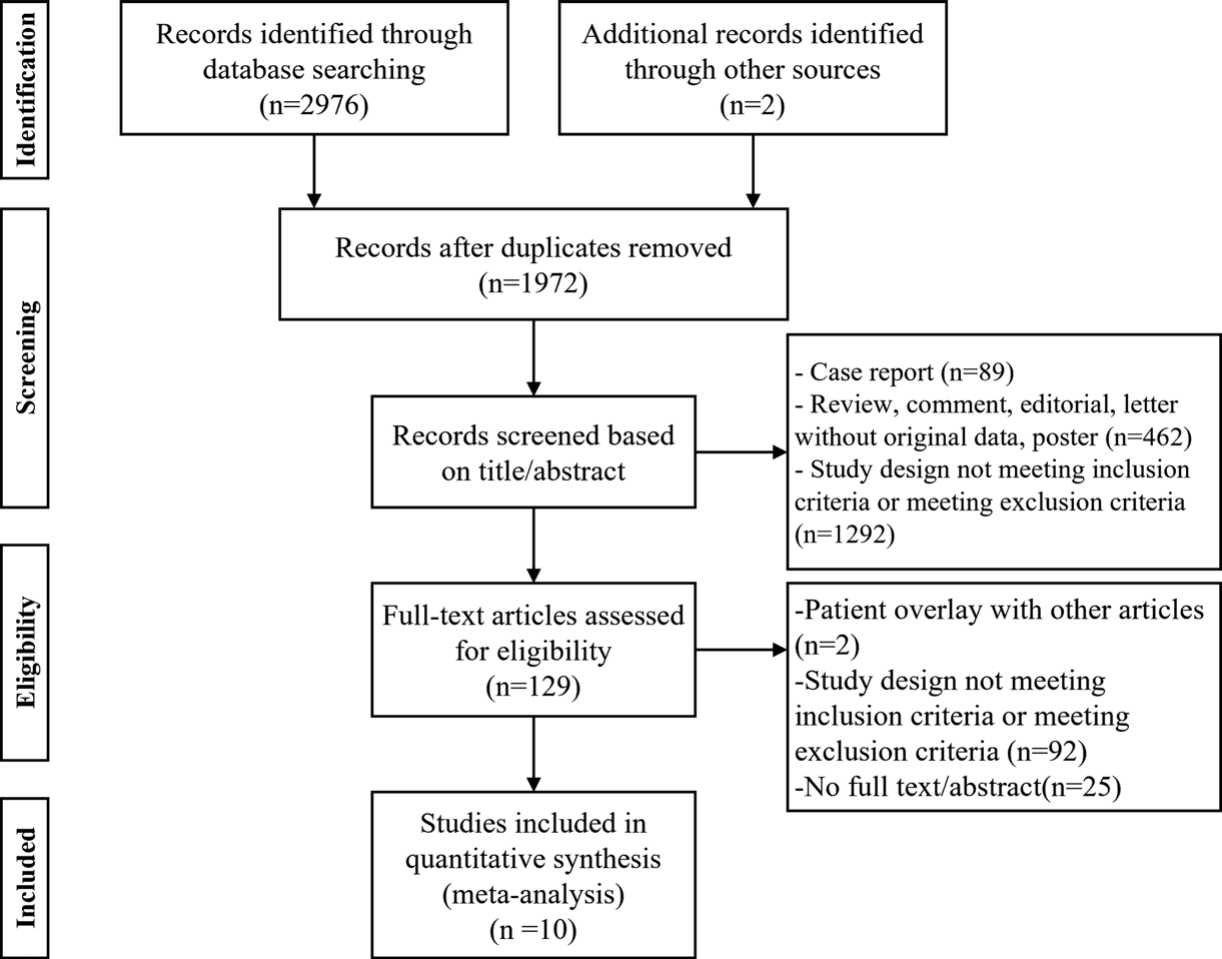
Systematic Reviews and Meta-Analyses (PRISMA) flow chart.

EndNote X9.1 (Version 19.1.0) was used to identify and remove duplicates. The remaining studies from the databases were filtered by their titles, abstracts, and keywords independently by two authors. Then, a comprehensive review of the studies’ full texts was conducted. If there was an overlap in research, the most recent and most extensive studies were selected for this meta-analysis. Ten studies were included in the final analysis. The PRISMA study selection flowchart is shown in Figure 1.

The inclusion criteria were as follows: (a) studies involved patients with a confirmed diagnosis of unresectable or metastatic iCCA and compared LRT + ST with ST treatment; and (b) studies reported clinical outcomes including any of the following: OS, progression-free survival (PFS), objective response rate (ORR) and adverse events (AEs). A study was excluded if it met any of the following criteria: (a) nonhuman studies; (b) population-level studies; (c) inadequate description of materials and methods; (d) raw data unavailable (letters, editorials, conference abstracts, posters, commentaries, and reviews); (e) clinical outcomes not reported for LRT + ST or ST; and (f) studies included patients with all types of cholangiocarcinoma and did not distinguish the clinical outcomes of patients with iCCA.

### Quality assessment

The studies were evaluated according to the Newcastle‒Ottawa Scale standards for cohort studies.^19^ Two authors independently assessed the quality of the studies, and any disagreements were resolved by discussion and consensus with a third author. Three columns comprise the scale: selection, comparability, and outcome. The score is rated out of 9 stars: >6 stars indicate a low risk of bias, 4–6 stars indicate a moderate risk of bias, and <4 stars indicate a high risk of bias. Out of the ten studies included^20,21,22,23,24,25,26,27,28,29^ in the systematic review, three studies^24,25,26^ were determined to be of good quality, and seven studies^20,21,22,23,27,28,29^ were determined to be of acceptable quality. Details of the quality assessment are shown in Supplementary Table 2.

### Data acquisition

The extracted primary information was as follows: (a) basic information, such as title, first author, journal, country or region, and publication date; (b) baseline characteristics of the study population, including sample size, age, gender, duration of follow-up, physical status, etc.; (c) interventions, including the type of medication, measurement, periodicity, frequency, etc.; (d) observed outcome data, including OS, PFS, ORR, AEs, etc.; (e) elements related to the inclusion and exclusion criteria; and (f) elements of the risk of bias evaluation. If the required information, such as the hazard ratio (*HR*) and 95% confidence interval (*95% CI*), were provided in the article, they were extracted directly; otherwise, they were calculated using the digital tools in Tierney's article.^30^ Engauge Digitizer (version 11.1) was used for digitizing the survival curves and then transforming the digital information to obtain the *HR* and *95% CI*. Two authors independently extracted data from the papers, and any discrepancies were resolved through a consensus meeting.

### Statistical analysis

Meta-analysis was only performed when data from at least three studies were available. StataMP 17 (Version 521.17.0.104) was used to conduct a meta-analysis. OS and PFS are time-to-event data evaluated by the *HR*. The ORR and AEs are enumeration data evaluated by the relative risk (*RR*). The *95% CI* was used for interval estimation. Heterogeneity between studies was analyzed by Cochran's Q test with a significance level of *α* = 0.1, and the degree of heterogeneity was assessed using *I*^2^ statistics. If *p* ≥ 0.10 and *I*^2^ ≤ 50%, heterogeneity was considered minor, and a fixed-effects model was used for analysis. Heterogeneity was considered major if *p* < 0.10 or *I*^2^ > 50%. The random-effects or fixed-effects model and sensitivity analysis were used to evaluate the stability of the outcome and identify articles with high heterogeneity. When necessary, subgroup analysis was performed. A *P value* < 0.05 was considered statistically significant.

## Results

### Baseline characteristics

Ten cohort studies were deemed eligible and included in the descriptive analysis; of the 3,791 patients in these studies, 1,120 (29.5%) were treated with LRT + ST. ADT was performed in six articles^20,22,23,24,27,28^, EBRT in three^25,26,29^, and ablation in one^21^ (Table 1). Further details of the included literature are provided in Supplementary Table 3.

**TABLE 1.** Main study characteristics of included studies

**Study**	**Country**	**Treatment**			**Sample**		**Age (years)**		**Sex (F:M)**		**Outcomes**

		**LRT + ST**		**ST**	**LRT + ST**	**ST**	**LRT + ST**	**ST**	**LRT + ST**	**ST**	
Yang, 2022^20^	China	**ADT: DEB-TACE (Doxorubicin)**	ICIs (Camrelizumab or Sintilimab)	Gemcitabine + Cisplatin	20	20	59	59	9:11	7:13	OS, PFS, ORR, AEs
Yan, 2022^21^	China	**Ablation: RFA / MWA**	Gemcitabine^*^	Gemcitabine^*^	36	36	NR	NR	14:22	15:21	OS
Sun, 2021^22^	China	**ADT: TACI (5-Fluoruracil + cisplatin)**	Gemcitabine + S-1	Gemcitabine + Cisplatin + S-1	33	33	NR	NR	NR	NR	OS, PFS, ORR, AEs
Gairing, 2021^23^	Germany	**ADT: TACE (Mitomycin C / Doxorubicin)**	Gemcitabine^*^	Gemcitabine^*^	14	59	61.3	66.8	8:6	29:30	OS
Hu, 2020^24^	China	**ADT: DEB-TACE (Gemcitabine + Cisplatin) / cTACE (Gemcitabine + Cisplatin + lipiodol)**	Apatinib	Apatinib	13	10	55.9	58.7	7:6	2:8	OS, PFS, ORR, AEs
Verma, 2018^25,&^	America	**EBRT**	SYS	SYS	666	2176	65	65	309:357	1095:1081	OS
Chang, 2018^26,&^	China	**EBRT: CCRT / CTRT**	Fluoropyrimidine^*^ / Gemcitabine^*^	Fluoropyrimidine^*^ / Gemcitabine^*^	211	211	60.11	60.80	81:130	84:127	OS
Konstantinidis, 2016^27^	America	ADT: HAI (Floxuridine^*^)	Gemcitabine^*^ / Irinotecan^*^ / 5-Fluoruracil^*^	Gemcitabine^*^ / 5-Fluoruracil^*^	78	26	62	26	47:31	13:13	OS, ORR
Edeline, 2015^28,&^	France	**ADT: ^90^Y SIRT**	Gemcitabine^*^ / 5-Fluoruracil^*^	Gemcitabine + Cisplatin^#^	24	33	NR	NR	NR	NR	OS, PFS
Kim, 2013^29^	Korea	**EBRT: CCRT**	Capecitabine + Cisplatin	Capecitabine + Cisplatin	25	67	56	58	6:19	14:53	OS, PFS, ORR, AEs

ADT = arterially directed therapy; AEs = adverse events; CCRT = concurrent chemoradiation therapy; cTACE = conventional transcatheter arterial chemoembolization; CTRT = sequential chemotherapy and radiotherapy; DEB-TACE = TACE with drug-eluting beads; ^90^Y SIRT = Yttrium-90 selective internal radiotherapy; EBRT = external beam radiation therapy; F = female; HAI = hepatic arterial infusion; ICIs = immune checkpoint inhibitors; LRT + ST = locoregional therapy combined with systemic therapy; M = male; MWA = microwave ablation; NR = not reported; ORR = objective response rate; OS = overall survival; PFS = progression-free survival; RFA = radiofrequency ablation; ST = systemic therapy; SYS = systemic chemotherapy; TACI = transarterial chemoinfusion; TACE = transarterial chemoembolization;

* = major drugs in the treatment regime;

# = data from the ABC-02 study;

& = multi-canter study.

All studies included were retrospective cohort studies.

### OS

**FIGURE 2.**
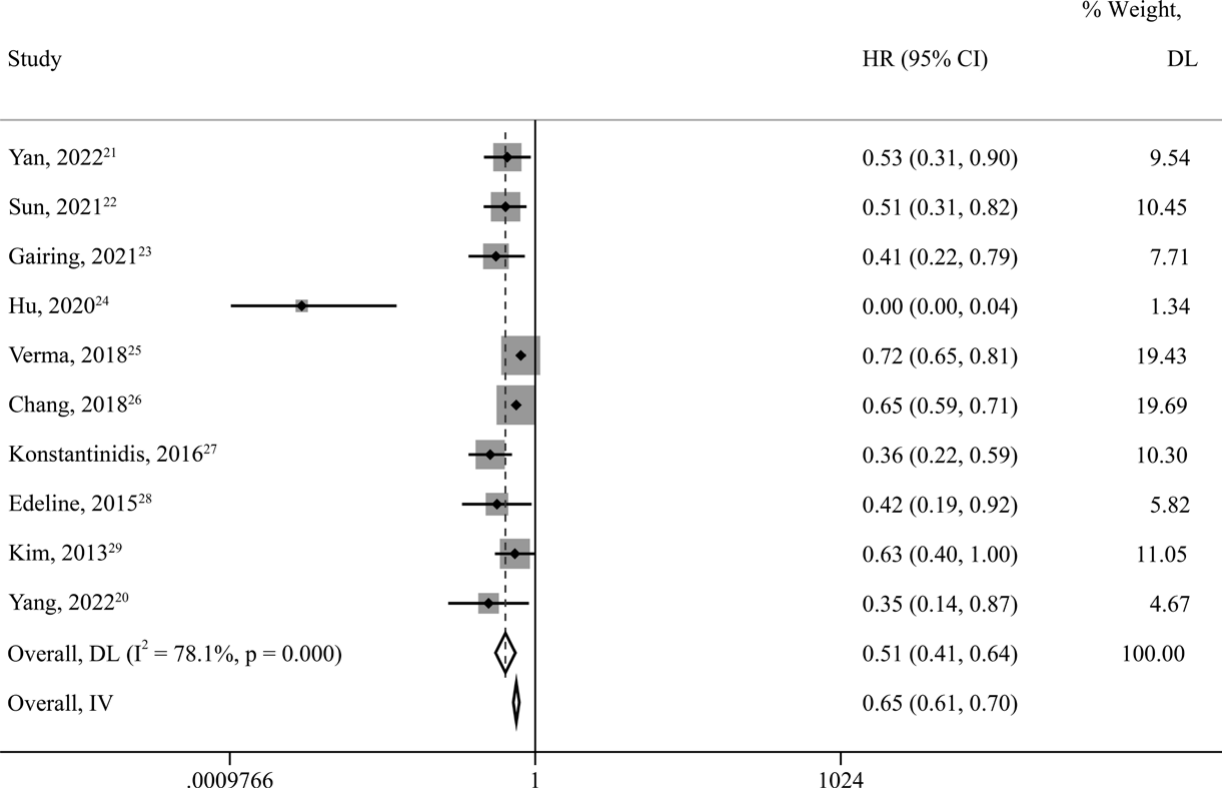
Forest plots for overall survival (OS) in unresectable and metastatic intrahepatic cholangiocarcinoma (iCCA) patients treated by locoregional therapy combined with systemic therapy versus only systemic therapy. 95% CI = 95% confidence intervals; DL = DerSimonian–Laird method; HR = hazard ratio; IV = inverse variance method

**FIGURE 3.**
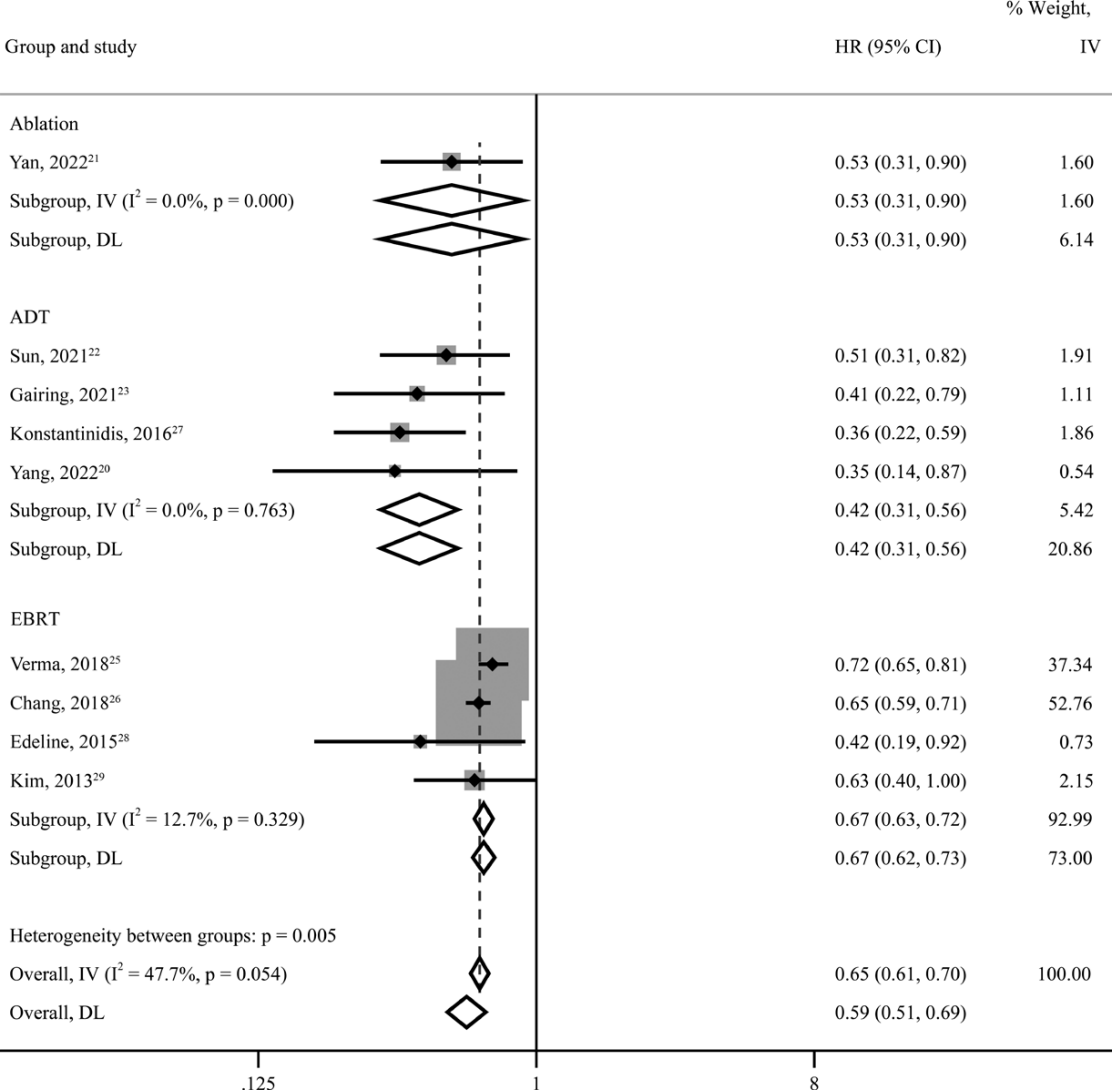
Subgroup analysis of overall survival (OS) in unresectable and metastatic intrahepatic cholangiocarcinoma (iCCA) patients according to types of locoregional therapy combined with systemic therapy (ablation, ADT, RT). 95% CI = 95% confidence intervals. ADT = arterially directed therapy; DL = DerSimonian–Laird method; HR = hazard ratio; IV = inverse variance method; EBRT= external beam radiation therapy

A total of 3,791 patients from all ten studies were included in the meta-analysis. The pooled *HR* indicated that compared with ST, LRT + ST highly significantly improved OS (*HR* = 0.51; *95% CI* = 0.41–0.64; *p value* < 0.001), reducing the risk of death by 49%. High heterogeneity existed among the ten studies (*I*^2^=78.1%, *p* < 0.001). Changing to a fixed-effects model for *HR* pooling showed that the outcome remained stable (*HR* = 0.65; *95% CI* = 0.61–0.70; *p value* < 0.001) (Figure 2). Sensitivity analysis was then performed and showed that the pooled *HR* was still reliable after deleting any of the articles (Supplementary Figure 1). Subgroup analysis according to locoregional therapies showed high heterogeneity in the ADT group (Supplementary Figure 2). Based on the above analysis, in addition to locoregional treatment, the heterogeneity mainly came from Hu's article^24^, which may be related to the intervention (apatinib) they adopted. When this article was removed, the heterogeneity was markedly reduced (*I*^2^ = 47.7%, *p* = 0.054). Subgroup analysis of the remaining nine articles showed that ST combined with ADT (*HR* = 0.42, *95% CI* = 0.31–0.56, *p value* < 0.001) or EBRT (*HR* = 0.67, *95% CI* = 0.63–0.72, *p value* < 0.001) improved patients’ OS and reduced the risk of death by 58% or 33% (Figure 3).

### PFS

**FIGURE 4.**
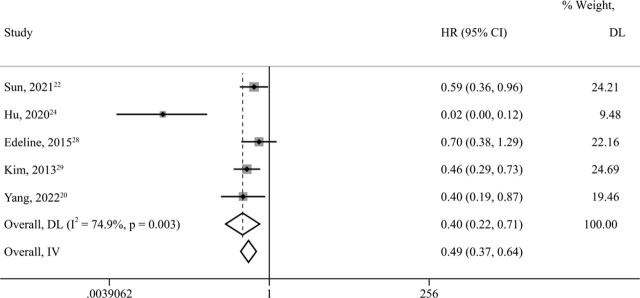
Forest plots for progression-free survival (PFS) in unresectable and metastatic intrahepatic cholangiocarcinoma (iCCA) patients treated by locoregional therapy combined with systemic therapy versus only systemic therapy. 95% CI = 95% confidence intervals; DL = DerSimonian–Laird method; HR = hazard ratio; IV = inverse variance method

The data on PFS were obtained from five studies^20,22,24,28,29^ with 278 patients. Among them, 115 (41.4%) were treated with LRT + ST. LRT + ST reduced the risk of tumor recurrence and metastasis by 60% more than ST (*HR* = 0.40, *95% CI* = 0.22–0.71, *p value* = 0.002). The five studies had high heterogeneity (*I*^2^=74.9%, *p* = 0.003). A fixed-effects model showed that the outcome remained stable (*HR* = 0.49; *95% CI* = 0.37–0.64; *p value* < 0.001) (Figure 4). Sensitivity analysis showed that the pooled *HR* was still reliable after deleting any of the articles (Supplementary Figure 3). Considering that the primary source of heterogeneity was still Hu's article^24^, the heterogeneity almost completely disappeared after omitting it (*I*^2^=0.0%, *p* = 0.609) (Supplementary Figure 4).

### ORR

**FIGURE 5.**
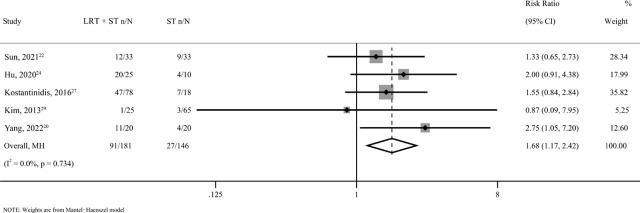
Forest plots for objective response rate (ORR) in unresectable and metastatic intrahepatic cholangiocarcinoma (iCCA) patients treated by locoregional therapy combined with systemic therapy (LRT + ST) versus only systemic therapy (ST). 95% CI = 95% confidence intervals; MH = Mantel–Haenszel model; RR = relative risk.

Five studies^20,22,24,27,29^ with 327 patients were used to analyze the ORR. 181 (55.4%) were treated with LRT + ST. The ORR of the LRT + ST group was better than that of the ST group (*RR* = 1.68, *95% CI* = 1.17–2.42, *p value* = 0.005) (Figure 5).

### AEs

**FIGURE 6.**
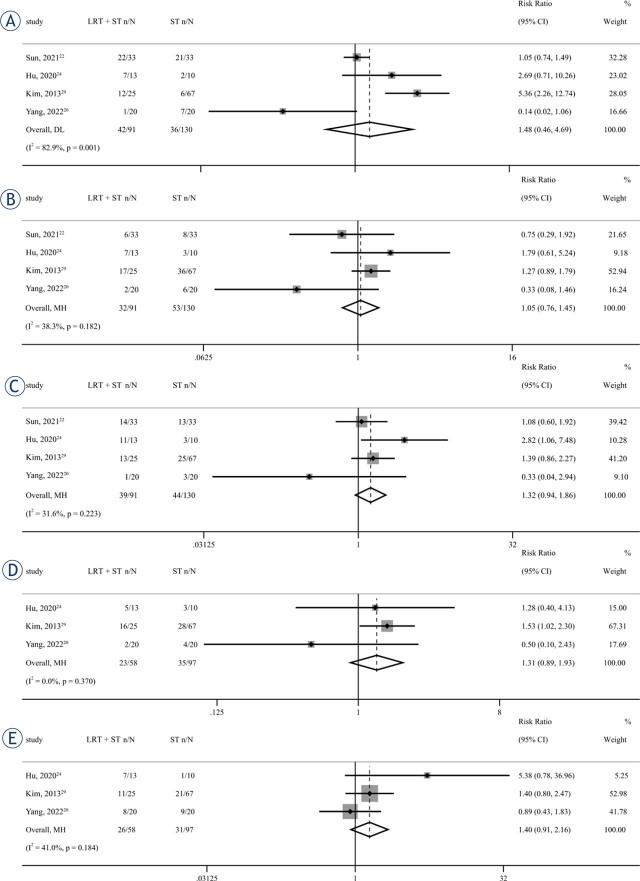
Forest plots for the incidence of neutropenia **(A)**, thrombocytopenia **(B)**, anemia **(C)**, anorexia **(D)**, and vomiting **(E)** in unresectable and metastatic intrahepatic cholangiocarcinoma (iCCA) patients treated by locoregional therapy combined with systemic therapy (LRT + ST) versus only systemic therapy (ST). 95% CI = 95% confidence intervals; DL = DerSimonian–Laird method; MH = Mantel–Haenszel model; RR = relative risk.

Four articles^20,22,24,29^, comprising a total of 221 patients, reported data on the occurrence of post-treatment neutropenia, thrombocytopenia, and anemia. Among these patients, 91 individuals (70.0%) received LRT + ST. Additionally, three articles^20,24,29^, involving 155 patients, presented data on post-treatment anorexia and vomiting, with 58 patients (37.4%) receiving LRT + ST. Neutropenia (*RR* = 1.48, *95% CI* = 0.46–4.69, *p value = 0.509*), thrombocytopenia (*RR* = 1.05, *95% CI* = 0.76–1.45, *p value* = 0.763), anemia (*RR* = 1.32, *95% CI* = 0.94–1.86, *p value* = 0.112), anorexia (*RR* = 1.31, *95% CI* = 0.89–1.93, *p value* = 0.167), and vomiting (*RR* = 1.40, *95% CI* =0.91–2.16, *p value* = 0.130) did not show significant differences between the LRT + ST group and ST group (Figure 6). Furthermore, among the four studies investigating treatment-related AEs, no instances of severe AEs, such as acute portal vein thrombosis, bleeding, biloma, abscess formation, bone marrow suppression, or pancreatitis, were observed. There were no reported cases of AEs-related death. More detailed data on AEs are recorded in Supplementary Table 4.

## Discussion

For unresectable or metastatic iCCA, the primary recommendation is GemCis or GemCis plus durvalumab. Additionally, combination or monotherapy regimens based on fluorouracil, capecitabine, and gemcitabine are also recommended. Entrectinib, larotrectinib, pembrolizumab, and pralsetinib may be used as first-line drugs for patients with specific gene expressions or immunohistochemical phenotypes. Besides these ST options, the current National Comprehensive Cancer Network® (NCCN®) guidelines also recommend locoregional therapies or LRT + ST to treat unresectable or metastatic iCCA.^31^ According to previous studies, the median OS for advanced iCCA patients who were treated with ST was 5.2 to 15.4 months.^32,33,34,35,36,37,38,39^ The emergence of locoregional therapies may bring more survival benefits to patients with unresectable or metastatic iCCA.

Locoregional therapies, including ablation, ADT, and EBRT, have emerged as promising treatments for iCCA. Ablation is widely used for single tumors smaller than 3 cm in nonsurgical candidates with iCCA.^7,8^ ADT can increase the local concentration of chemotherapeutic drugs while minimizing systemic adverse effects.^10,11,12,13^ EBRT, especially combined with standard or high-dose fluorouracil chemotherapy, has been recommended by the NCCN guidelines as one of the standard treatments for locally advanced iCCA.^14,17,40^ However, the current NCCN guidelines lack specific recommendations regarding the treatment approach for locoregional therapies in combination with systemic therapy, aside from chemoradiotherapy, due to insufficient evidence-based medicine.

This systematic review and meta-analysis evaluated the clinical outcomes of LRT + ST and ST using data from 10 cohort studies with 3,791 patients. The OS and PFS in the LRT + ST group were much better than those in the ST group, and the ORR was improved. Subgroup analysis based on the type of locoregional therapy indicated that the combination of ST with ADT or EBRT might increase the OS of patients and lower their risk of death. The sole trial of ST combined with ablation^21^ showed that LRT + ST reduced the risk of death by 47%. Particularly worth mentioning is that Hu's study^24^ was highly heterogeneous among the ten included articles, which is likely attributed to the use of apatinib as a targeted drug rather than the first-line chemotherapy primarily recommended in the guidelines. Apatinib is a novel, small molecule, selective vascular endothelial growth factor receptor-2 (VEGFR-2) tyrosine kinase inhibitor and has been confirmed to be effective in various advanced cancers including gastric cancer, hepatocellular carcinoma, non-small cell lung cancer, and breast cancer.^41,42,43,44^ In biliary tract cancer, apatinib is also in the clinical exploration stage and has not yet been listed as standard treatment for iCCA in guidelines.^45,46,47^

Complete resection is the only potential curative treatment for iCCA, whereas systemic therapy and locoregional treatments are considered palliative measures for patients diagnosed with unresectable or metastatic disease. Notably, in Konstantinidis’ study, eight patients initially deemed unresectable iCCA underwent curative-intent surgical resection. Among these cases, four patients received systemic chemotherapy, three underwent systemic chemotherapy combined with hepatic arterial infusion (HAI), and one received isolated HAI. Their postoperative median overall survival was 36.9 months (range: 10.4–92.3 months).^27^ In the Phase II single-arm MISPHEC trial, the combination of radioembolization with Y-90 microspheres with GemCis served as a first-line treatment approach for 41 unresectable iCCA patients, resulting in tumor downstaging and subsequent surgery for nine patients (22%).^10^ Currently, the NCCN guidelines do not provide recommendations for neoadjuvant/conversion therapy strategies for unresectable/metastatic iCCA. However, the integration of LRT + ST may represent a promising direction for achieving downstaging to resection of initially unresectable tumors in the future.

In addition to evaluating the effectiveness of LRT + ST, this study focused on the occurrence of AEs. In this study, four studies^20,22,24,29^ reported the incidence of complications. Because few studies provided the same AE outcomes, meta-analyses were only performed on neutropenia, thrombocytopenia, anemia, anorexia, and vomiting. It was confirmed that LRT + ST did not increase the incidence of AEs compared with ST, consistent with the results of the four studies included. Particularly worth mentioning is a statistically significant difference in the incidence of neutropenia between the two groups in Yang's and Kim's studies. However, in Yang's study, the incidence in the ST group was higher, while in Kim's study, the incidence in the LRT + ST group was higher, which may be related to the different interventions they used. Both Yang's and Kim's ST groups were treated with GemCis, but Kim's experimental group was treated with concurrent chemoradiation therapy, while Yang's experimental group was treated with transarterial chemoembolization with drug-eluting beads (DEB-TACE) + immune checkpoint inhibitors (ICIs). In addition, in Kim's study, the incidence of hand-foot syndrome was higher in the LRT + ST group. In Yang's study, the incidences of leukopenia, hypothyroidism, and reactive cutaneous capillary endothelial proliferation (RCCEP) were significantly different between the two groups, but this may be due to the use of ICIs. In conclusion, based on the scant data available, there is no solid evidence that LRT + ST will bring an additional AE burden to iCCA patients.

This meta-analysis study also had several limitations. First, the LRT + ST used in the study was diverse and nonstandard treatment. Among the ten original studies incorporated, there were variations in the selection of locoregional treatment modalities, the timing of incorporating locoregional therapies, and the local pharmacological interventions for interventional therapy. Future guidelines should specify when and what locoregional therapies should be combined. Second, the application of chemotherapy schemes included in the study was not completely consistent, which led to strong heterogeneity between different studies and the failure to obtain a specific survival time. Extensive randomized controlled trials are needed to confirm the findings of this study.

In conclusion, LRT + ST resulted in more survival benefits than ST without increasing the incidence of complications for unresectable and metastatic iCCA, which can be used as a supplement to the practice guidelines.
